# Comparative perspectives: a cross-sectional survey of key stakeholders regarding OB/GYN accessibility for women with disabilities

**DOI:** 10.3389/fpubh.2026.1812493

**Published:** 2026-06-26

**Authors:** Ashley E. Hilton, Cody Unser, Rishika Kartik, Ashira Greenberg, Amy A. Ewing, Murphy D. Kaphing, Lizbeth Grado, Jeanelle Sheeder, Marsha K. Guess

**Affiliations:** 1 Division of Urogynecology and Reconstructive Pelvic Surgery, Department of Obstetrics and Gynecology, University of Colorado School of Medicine, Aurora, CO, United States; 2 Department of Sociology of Health and Illness, University of New Mexico, Albuquerque, NM, United States; 3 Independent Researcher, New York, NY, United States; 4 Department of Maternal and Child Health, University of Colorado School of Public Health, Aurora, CO, United States; 5 Division of Complex Family Planning, Department of Obstetrics and Gynecology, University of Colorado School of Medicine, Aurora, CO, United States

**Keywords:** disability, gynecology, healthcare accessibility, healthcare disparities, obstetrics

## Abstract

**Introduction:**

An estimated 1.3 billion people and over one-fifth of women globally live with a disability (1). Women with disabilities face lower rates of preventative care and higher rates of morbidity and premature mortality compared to persons without a disability. Obstetrics and gynecology care providers (OB/GYN providers) deliver longitudinal care to women across the lifecycle and serve as important stakeholders for bringing clarity and context to conversations about health care accessibility for patients with disabilities (patients), while patients and their advocates are critical contributors, to ensure that information obtained reflects lived experiences. The aim of this study was to compare the experiences and perceptions of patients, OB/GYN providers, and advocates regarding accessibility of OB/GYN care.

**Methods:**

A quantitative, descriptive, cross-sectional study was conducted using an online survey distributed to patients, OB/GYN providers, and advocates through professional networks, advocacy groups, and social media from August 2024–April 2025. Surveys gathered demographic data as well as perceptions of barriers and facilitators to care and related outcomes. Thematic analysis of open-ended questions was also conducted.

**Results:**

A total of 369 participants completed surveys. Most patients had physical (74.4%) or cognitive disabilities (48.2%). Compared to patients, OB/GYN providers and advocates were more likely to report care delays (23.5 vs. 50.7 vs. 30.8%) and rescheduled appointments (18.0 vs. 22.1 vs. 19.4%) and less likely to report appointment cancellations (15.7 vs. 10.2 vs. 12.9%), due to unmet accommodation needs (all *p* < 0.001). Patients reported wait times of 1 month to 1 year to have a canceled appointment rescheduled; many OB/GYN providers (62.5%) reported rescheduling wait times of < 2 weeks. Most patients (86.7%) reported not receiving needed care when their appointments were canceled without rescheduling, while 14.3% of OB/GYN providers and 50.0% of advocates reported that patients never received care (*p* < 0.001). Nearly all care providers (94.2%) believed that OB/GYN teams make the effort to prioritize comfort and safety for disabled patients. One-third of providers were unaware of what accommodations their offices provided. Patients identified physical barriers (51.9%) and social barriers (29.2%) as primary factors negatively impacting care in both questionnaire and open-ended responses.

**Discussion:**

Discordant perceptions exist between patients, OB/GYN providers and advocates regarding the prevalence and impact of disruptions in OBGYN care for patients with disabilities and OB/GYN provider sensitivity and care quality provided to affected patients. Engaging key stakeholders with different and valuable perspectives in conversations helps establish a critical framework from which to develop harmonious solutions that can reduce care gaps and improve OBGYN care for patients with disabilities.

## 1 Introduction

Among the approximately four billion women worldwide, an estimated 700 million women and girls (19%) have disabilities (1–3). Obstetrics and gynecology (OB/GYN) care providers deliver longitudinal primary, maternal, gynecologic and surgical care to women, allowing them to build long-term relationships across the lifecycle. Despite efforts to improve accessibility in general medical settings, obstetrics and gynecology (OB/GYN) has seen less progress, with many offices remaining inaccessible and unprepared to address the complex needs of patients with disabilities (4). Women with disabilities are twice as likely to have sexually transmitted infections and face 11 times the risk of maternal death compared to women without disabilities (4, 5). Rates of sexual violence are three times higher for women with disabilities (6, 7); however, access to trauma-informed reproductive healthcare remains limited. Cancer screening disparities are equally concerning, with women with disabilities 22% less likely to receive regular breast cancer screening—disparities that reach 37% lower rates for women with visual impairments (8–11). Women with disabilities are also significantly less likely to receive cervical cancer screening within the recommended timeframes (12–14).

OB/GYN care presents many challenges for patients with disabilities. The specialty involves intricate procedures and sensitive discussions that require specialized communication skills and cultural competency (15). Physical examinations often require patients to undress and assume challenging positions that can be painful or impossible without proper accommodation and support (16). Identifying barriers and facilitators of accessibility is critical to the establishment of successful interventions that can improve OB/GYN care for women with disabilities.

Patients offer a perspective from lived experience, while providers operate within systems to provide care to patients throughout the lifespan and advocates observe and help facilitate care from a relational vantage point. Capturing all three perspectives is essential to identifying the full scope of barriers and facilitators of accessibility for patients, to understand their perceived impact on patient care and to catalyze change. The aim of this study was to compare the experiences and perceptions of patients with disabilities, care providers, and advocates regarding accessibility of OB/GYN care for patients with disabilities using a tri-stakeholder cross-sectional survey design.

## 2 Materials and methods

A quantitative, descriptive, cross-sectional study was conducted using an online survey co-designed by a team of researchers at an academic medical center (AH and MKG), disability advocates and community experts with lived experience of disability (CU, AG and AE), and community members participating in disability advocacy work (RK). The inclusion criteria were age ≥18 years and self-designation as a representative of one of the following three groups: an individual with a disability (patient), a provider of OB/GYN care to patients (care providers), or an advocate of a person with a disability (advocate), defined as a family member or significant other of a person with a disability or an employed person or community volunteer focused on disability matters. Exclusion criteria included individuals under the age of 18, those who did not identify with any of the three participant groups, and individuals who had no direct experience delivering, receiving, or advocating for OB/GYN care. Participants were recruited using flyers distributed through OB/GYN offices, independent living centers, healthcare facilities, professional networks, disability advocacy groups and social media platforms from August 2024–April 2025. The study was approved for exemption by the Colorado Multiple Institutional Review Board (COMIRB; protocol #24-1484).

The survey was constructed based on a comprehensive literature review and input from experts in disability advocacy and healthcare accessibility. The instrument collected demographic information including the type and duration of disability for patients and advocates and the specialty, number of years in practice, and scope of training in disability care for providers. Participants were also queried about accommodations needed and received, the impact of missing accommodations on receiving care and perceptions of care quality. Survey validation was conducted with 15 participants, including patients with diverse disability types, care providers and advocates, to ensure accessibility, readability, and content validity. Reading level was assessed at Flesch-Kincaid grade level 7.3. Minor modifications to question wording and formatting were implemented based on feedback to enhance clarity and accessibility. Participants completed surveys electronically using QR codes or HTML links that directed them to a HIPAA-compliant REDCap survey database. The risk of survey questions having an emotional impact on participants when responding to sensitive topics or invoking negative feelings from reliving traumatic events was communicated to all participants, and support resources were provided.

For all questions, modified language was used to direct the question to the appropriate respondent. For example, if a participant self-designated as a patient or advocate they would receive a question that asked “Have you ever had an OB/GYN appointment canceled because the office could not accommodate your needs”, while providers received the question “Have you ever had to cancel an OB/GYN appointment for an individual with a disability because the office could not accommodate their needs?” Four questions were used to assess participants' subjective evaluation of the OB/GYN care quality provided to patients with disabilities (Supplemental 1). Responses were provided using a modified 5-point Likert scale that ranged from “strongly agree” to “strongly disagree.” The responses of “strongly agree” and “agree” were considered positive. For one question, assessing need for greater accessibility in OB/GYN care teams, reverse scoring was used and the responses “Neutral”, “Disagree” and “Strongly disagree” were considered positive.

The primary outcome was the proportion of individuals who reported modifying OB/GYN appointments (i.e., experiencing delays, rescheduling, or outright cancellations) due to a lack of accommodations. This outcome was measured by asking each participant group a corresponding dichotomous question (yes/no) about appointment modification attributable to unmet accommodation needs and was analyzed using chi-square comparisons across the three groups. Secondary outcomes included wait times for rescheduling appointments, the frequency of pre-appointment screening for disabilities, availability and adequacy of accommodations in OB/GYN offices, the sensitivity of the doctor and healthcare team to patients with disabilities, and factors that positively and negatively impact OB/GYN care for patients with disabilities.

This study used a convenience sample of patients, advocates and care providers. As the primary objective was descriptive—to characterize perspectives, experiences, and self-reported practices—no formal *a priori* sample size calculation was performed. We aimed to enroll a sample large enough to provide estimates of key factors within each respondent group. Because of the descriptive and exploratory nature of this study, summary statistics and *p*-values should be interpreted accordingly.

Descriptive statistics were used for demographic data and to analyze frequencies, central tendencies, and measures of variation. Subgroup analyses were conducted to explore differences in perceptions between patients, providers of OB/GYN care and advocates, and practitioners and their care teams, as well as across individuals with different types of disabilities. Open-ended questions were evaluated to to identify key factors that supplement quantitative findings and identify specific areas for improvement. Pearson chi-square tests with the Yates continuity correction were used for categorical variables and the Welch *t*-test for continuous data. All statistical analyses were performed using SAS Version 9.2. *P* < 0.05 was considered statistically significant.

## 3 Results

A total of 369 participants completed the surveys, including 195 individuals with disabilities (52.8%), 140 OB/GYN care providers (37.9%), and 34 advocates (9.2%) (Table 1). Most respondents across all groups identified as white females (87.2%; 95% CI: 82.5–91.9% of patients with disabilities, 85.7%; 95% CI: 79.9–91.5% of providers, and 94.1%; 95% CI: 86.2–100% of advocates). Fewer than 15%; 95% CI: 6.2–12.3% of participants in each group identified as Hispanic (Table 1). Participants significantly differed by education level, employment status and income (*p* < 0.05; Table 1). Educational attainment was generally high, with more than 70% of individuals in each group reporting a bachelor's degree or higher.

**Table 1:** Participant characteristics.

Demographics	Patients *N* = 195; 52.8%	Providers *n* = 140; 37.9%	Advocates *n* = 34; 9.2%	*P*
Age	38.2 (18–71)	38.2 (25–70)	35.9 (20–63)	0.85
Gender				
Female	170 (87.2%)	120 (85.7%)	32 (94.1%)	0.42
Race				
White	142 (74.3%)	110 (78.6%)	27 (79.4%)	0.64
Black	18 (9.4%)	12 (8.6%)	2 (5.9%)	
Asian	9 (4.7%)	10 (7.1%)	1 (2.9%)	
Pacific Islander	1 (0.5%)	0	0	
Native American	2 (1%)	0	0	
Two or more races	10 (5.2%)	6 (4.3%)	4 (11.8%)	
Other	4 (2.1%)	1 (0.7%)	0	
Decline	5 (2.6%)	1 (0.7%)	0	
Ethnicity				
Hispanic/Latin(x)	17 (8.9%)	11 (7.9%)	5 (14.7%)	0.53
Primary language spoken				
English	187 (96.9%)	136 (97.8%)	24 (100%)	0.48
Spanish	1 (0.5%)	2 (1.4%)	0	
Other	5 (2.6%)	1 (0.7%)	0	
Education level				
None or grades 1–11	0	0	0	< 0.001
High school/GED	15 (7.7%)	0	0	
Some college/technical school	36 (18.5%)	0	2 (5.9%)	
College graduate	68 (34.9%)	3 (2.1%)	18 (52.9%)	
Graduate/professional school	76 (39%)	137 (97.9%)	14 (41.2%)	
Employment status				
Full-time	70 (35.9%)	123 (87.9%)	24 (70.6%)	< 0.001
Part-time	39 (20%)	9 (6.4%)	6 (17.6%)	0.002
Unable to work due to disability	50 (25.6%)	0	1 (2.9%)	< 0.001
Out of work/looking for work	14 (7.2%)	2 (1.4%)	0	0.017
Out of work/not looking for work	3 (1.5%)	0	0	0.26
Homemaker	4 (2.1%)	0	0	0.17
Student	25 (12.8%)	4 (2.9%)	1 (2.9%)	0.002
Military	1 (0.5%)	3 (2.1%)	0	0.30
Retired	12 (6.2%)	1 (0.7%)	1 (2.9%)	0.04
Other	5 (2.6%)	0	0	0.10
Total household income				
Under $25,000	46 (23.7%)	1 (0.7%)	0	< 0.001
$25,000–$49,999	26 (13.4%)	0	3 (8.8%)	
$50,000–$74,999	31 (16%)	11 (8%)	5 (14.7%)	
$75,000–$99,999	24 (12.4%)	18 (13.1%)	6 (17.6%)	
$100,000–$149,999	26 (13.4%)	20 (14.6%)	6 (17.6%)	
$150,000 or more	41 (21.1%)	87 (63.5%)	14 (41.2%)	
Gravity				
0	117 (60%)	70 (50.4%)	19 (55.9%)	0.0.04
1	32 (16.4%)	14 (10.1%)	2 (5.9%)	
2	25 (12.8%)	18 (12.9%)	6 (17.6%)	
3	12 (6.2%)	21 (15.1%)	4 (11.8%)	
4	5 (2.6%)	6 (4.3%)	2 (5.9%)	
5+	4 (2.1%)	10 (7.2%)	1 (2.9%)	
Parity				
0	11 (14.1%)	9 (13.2%)	1 (6.7%)	0.05
1	30 (38.5%)	11 (16.2%)	2 (13.3%)	
2	28 (35.9%)	30 (44.1%)	10 (66.7%)	
3	7 (9%)	16 (23.5%)	2 (13.3%)	
4	2 (2.6%)	1 (1.5%)	0	
5+	0	1 (1.5%)	0	
Type of disability				
Physical/mobility	145 (74.4%)	–	–	–
Mental illness	58 (29.7%)	–	–	–
Developmental/learning	36 (18.5%)	–	–	–
Deaf/hard of hearing	16 (8.2%)	–	–	–
Blind/low vision	11 (5.6%)	–	–	–
Speech/language	10 (5.1%)	–	–	–
Other	12 (6.2%)	–	–	–
Type of provider				
Ob/Gyn physician	–	112 (80.0%)	–	–
Advanced practice provider	–	19 (13.5%)	–	–
Midwife	–	2 (1.4%)	–	–
Family medicine physician	–	1 (0.7%)	–	–
Other	–	7 (5.0%)	–	–
Number of years in practice				
Still in training	–	39 (34.2%)	–	–
0–5 years	–	17 (14.9%)	–	–
6–10 years	–	9 (7.9%)	–	–
10–20 years	–	19 (16.7%)	–	–
20 + years	–	30 (26.3%)	–	–
Type of practice				
Academic	–	82 (59.0%)	–	–
Hospital-based	–	38 (27.3%)	–	–
Private	–	11 (7.9%)	–	–
Community health clinic	–	5 (3.6%)	–	–
Other	–	3 (2.2%)	–	–

**Figure 1:**
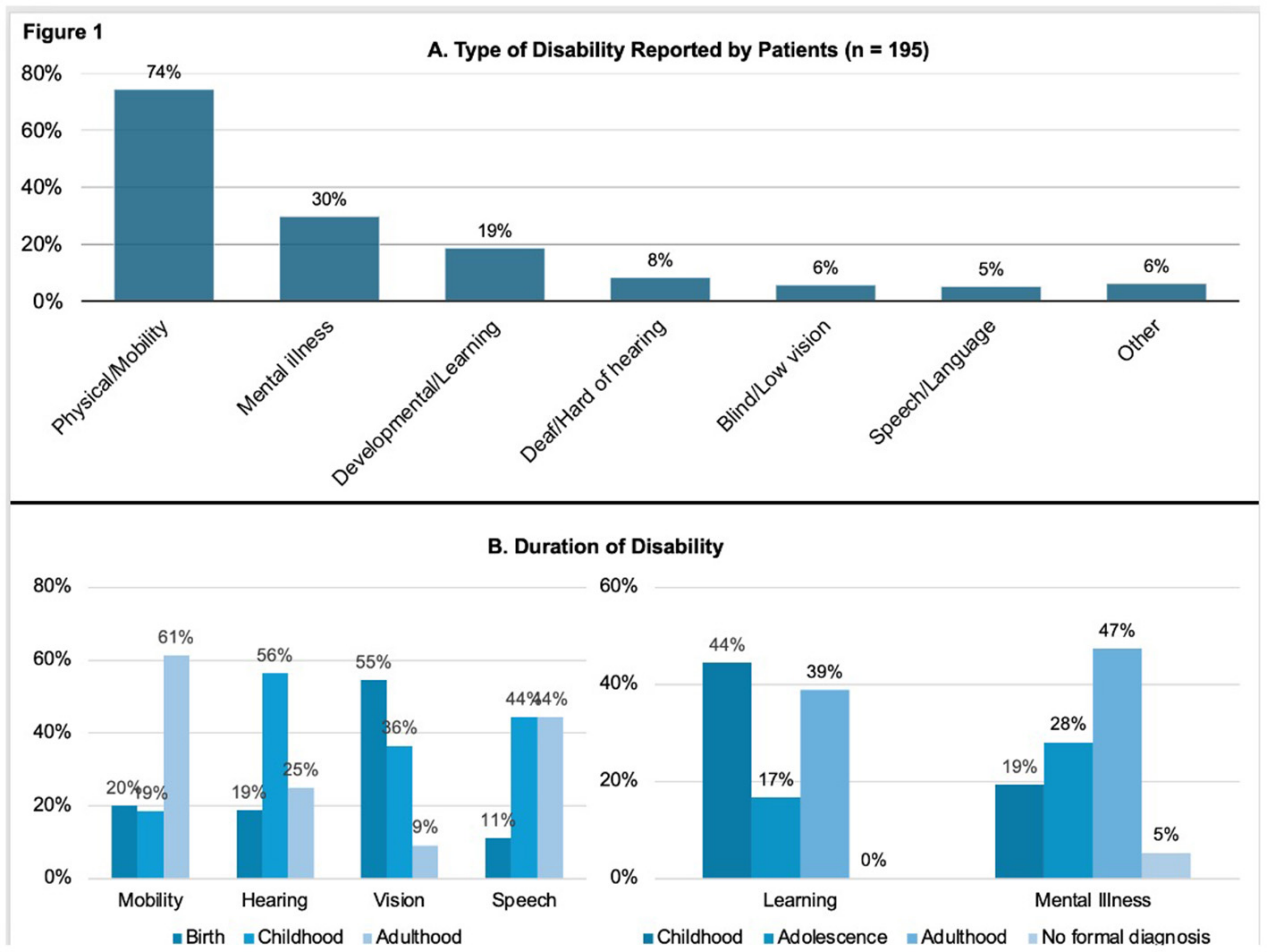
Disability characteristics of patient participants. **(A)** Distribution of disability types reported by patient participants (*n* = 195). Participants could select multiple disability types; percentages reflect the proportion of patients reporting each category. **(B)** Age of disability onset reported by patient participants. Physical and cognitive disabilities were most commonly reported, with the majority developing in adulthood.

**Figure 2:**
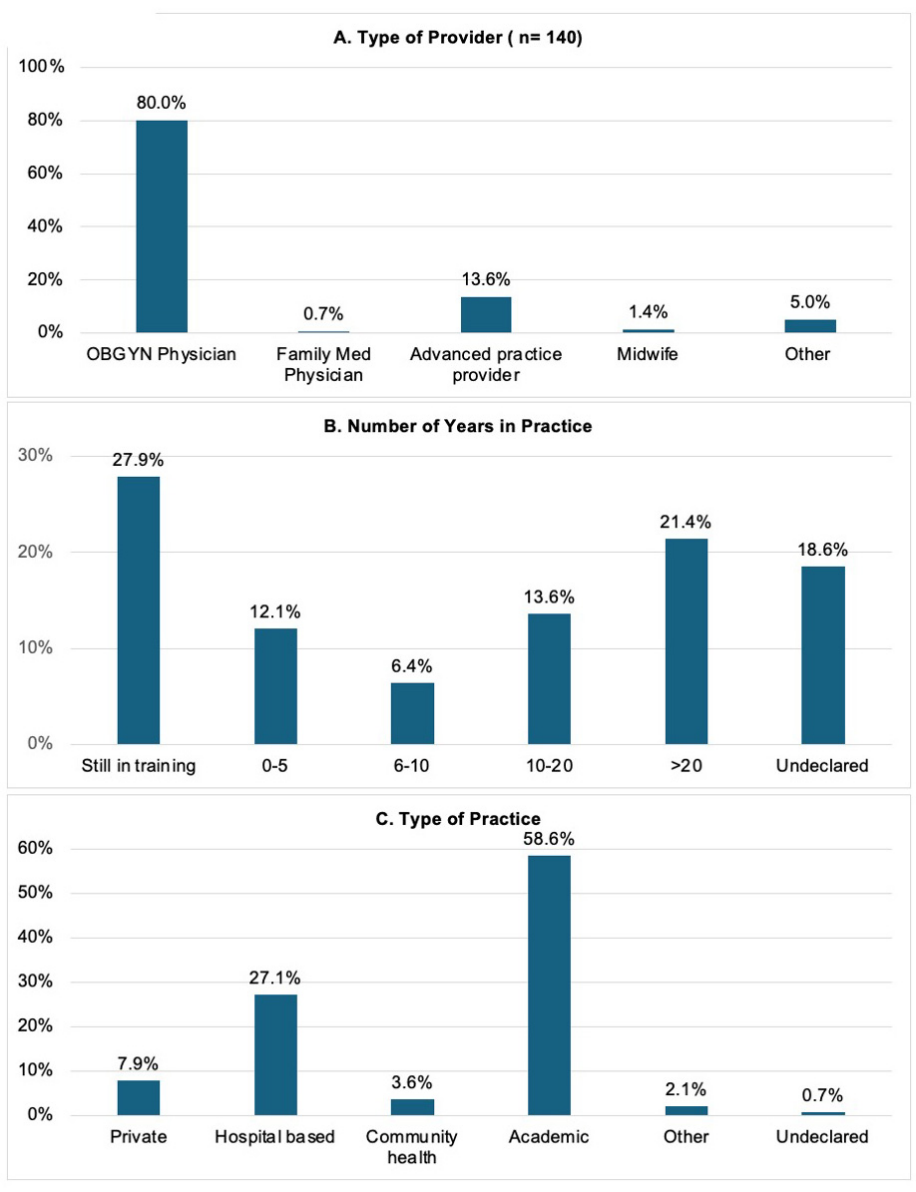
Characteristics of care provider participants. **(A)** Provider type among care provider participants (*n* = 140). The majority were OB/GYN physicians (79.4%), with the remainder comprising nurse practitioners, certified nurse midwives, and other OB/GYN care team members. **(B)** Training status of OB/GYN physician respondents; 27.9% were still in training at the time of the survey. **(C)** Practice setting of care provider participants; 59% were affiliated with academic medical centers, with generalists and subspecialists evenly represented (52.8% and 47.2%, respectively).

Patients most commonly reported physical (74.4%; 95% CI: 67.6–80.3%) and cognitive (mental illness and learning) disabilities (48.2%; 95% CI: 41.0–55.5%) that developed in adulthood (Figures 1A, B). Over one-third reported multiple types of disabilities. Nearly all patients (94.8%; 95% CI:90.8–97.5%) had previously visited an OB/GYN office or clinic. The majority of providers were OB/GYN physicians (79.4%; 95% CI: 71.6–85.7% Figure 2A), many of whom were still in training (34.2%; 95% CI: 26.5–42.7% Figure 2B) and affiliated with academic medical centers (59%; 95% CI: 50.7–67.5% Figure 2C). Generalists and subspecialists were evenly represented among the OB/GYN physician respondents (52.8%; 95% CI: 43.5–62.7 and 47.2%; 95% CI: 37.3–59.6%, respectively).

There were significant differences in perceptions regarding care disruptions due to an inability to accommodate disabilities (*p* < 0.001 for all comparisons; Table 2). Providers reported the highest rates of care delays due to unmet accommodation needs (50.7%; 95% CI: 41.4–58.6%), followed by advocates (30.8%; 95% CI: 17.4–50.5%) and patients (23.5%; 95% CI: 17.4–50.5%). Similarly, providers more frequently reported the need to reschedule appointments (22.1%; 95% CI 15.4–30.0 vs. 19.4%; 95% CI: 7.5–37.5% of advocates and 18.0%; 95% CI: 12.8–24.2% of patients). Patients reported wait times to reschedule were either ≤ 1 month (30%; 95% CI: 14.7–49.4%) or 6 months−1 year (33.3%; 95% CI: 17.3–52.8%). In contrast, nearly two-thirds of providers reported rescheduling patients within 2 weeks. Only two advocates responded to this question, reporting wait times of 1 month and 3–6 months, respectively (*p* = 0.097; Table 2). Patients were more likely to report cancellations due to lack of accommodations (15.7%; 95% CI: 10.9–21.7%) compared to providers (10.2%; 95% CI: 5.7–16.6%) and advocates (12.9%; 95% CI: 3.6–29.8%) (*p* < 0.001; Table 2). Eighty-six percent (95%CI: 69.3–96.2%) of patients who experienced cancellations reported not receiving care at all. Fewer providers (14.3%; 95% CI: 1.8–42.8%) and half of the advocates (50.0%; 95% CI: 6.8–93.2%) reported that care was never received following cancellations (*p* < 0.001; Table 2). Most patients (84.5%; 95% CI: 78.4–89.5%) reported that their accommodation needs were not assessed prior to their visit and over half of the providers (55.8%; 95% CI: 46.8–64.0%) stated they were unsure whether such assessments occurred at their offices. Less than 5% (95%CI: 0.1–19.6%) of advocates reported that accommodations were proactively assessed in advance of the visit (*p* < 0.001; Table 2).

**Table 2:** Comparison of responses.

Assessments	Patients	Providers	Advocates	*P*
Have you ever had a delay in your services because an OB/GYN office did not have needed accommodations readily available?				
Yes	43 (23.5%)	70 (50.7%)	8 (30.8%)	< 0.001
No	118 (64.5%)	48 (34.8%)	17 (65.4%)	
Don't know	22 (12%)	20 (14.5%)	1 (3.8%)	
How long was the delay?				
Less than 30 min	14 (33.3%)	25 (35.7%)	1 (14.3%)	0.049
30 min to an h	10 (23.8%)	29 (41.4%)	1 (4.3%)	
1–2 h	10 (23.8%)	10 (14.3%)	4 (57.1%)	
>2 h	8 (19%)	6 (8.6%)	1 (14.3%)	
**Have you ever had an OB/GYN appointment rescheduled because an OB/GYN office could not accommodate your (or your patient's) needs?**				
Yes	34 (18%)	30 (22.1%)	6 (19.4%)	< 0.001
No	147 (77.8%)	69 (50.7%)	22 (71%)	
Don't know	8 (4.2%)	37 (27.2%)	3 (9.7%)	
**Approximately how long did you (or your patient) wait for the next appointment?**				
< 1 week	1 (2.9%)	3 (10%)	0	< 0.001
1–2 weeks	6 (17.6%)	13 (43.3%)	1 (20%)	
1 month	2 (5.9%)	6 (20%)	1 (20%)	
3–6 months	11 (32.4%)	1 (3.3%)	0	
6 months−1 year	7 (20.6%)	1 (3.3%)	1 (20%)	
>1 year	3 (8.8%)	0	0	
**Have you ever had an OB/GYN appointment canceled because the office could not accommodate your (or your patient's) needs?**				
Yes	30 (15.7%)	14 (10.2%)	4 (12.9%)	< 0.001
No	151 (79.1%)	76 (55.5%)	24 (77.4%)	
Don't know	10 (5.2%)	47 (34.3%)	3 (9.7%)	
**Was your (or your patients) appointment rescheduled?**				
Yes	4 (13.3%)	8 (57.1%)	2 (50%)	< 0.001
No, did not receive care	26 (86.7%)	2 (14.3%)	2 (50%)	
Don't know	0	4 (28.6%)	0	
Approximately how long did you (or your patient) wait for the next appointment?				
< 1 week	0	1 (12.5%)	0	0.10
1–2 weeks	0	4 (50%)	0	
1 month	2 (50%)	2 (25%)	1 (50%)	
0.103–6 months	0	0	1 (50%)	
6 months−1 year	2 (50%)	0	0	
Don't know	0	1 (12.5%)	0	
Did your OB/GYN office ask about your (or your patients) need for accommodations prior to the visit?				
Yes	19 (10.5%)	–	1 (3.8%)	–
No	153 (84.5%)	–	24 (92.3%)	–
Don't know	9 (5%)	–	1 (3.8%)	–
**Positive responses on likert scale:**	**Patients**	**Providers**	**Advocates**	***P***
**Effort to prioritize comfort and safety**				
Agree/strongly agree	88 (48.4%)	131 (94.2%)	9 (36%)	< 0.001
Feeling heard and understood				
Agree/strongly agree	79 (43%)	132 (95%)	6 (24%)	< 0.001
Perception of accommodations				
Agree/strongly agree	47 (25%)	64 (46%)	4 (12.9%)	< 0.001
Need for greater accessibility				
Neutral/disagree/strongly disagree	39 (20.4%)	18 (12.9%)	2 (6.5%)	0.06

Pearson chi- square test was used. Values are significant where *P* < 0.05.

**Figure 3:**
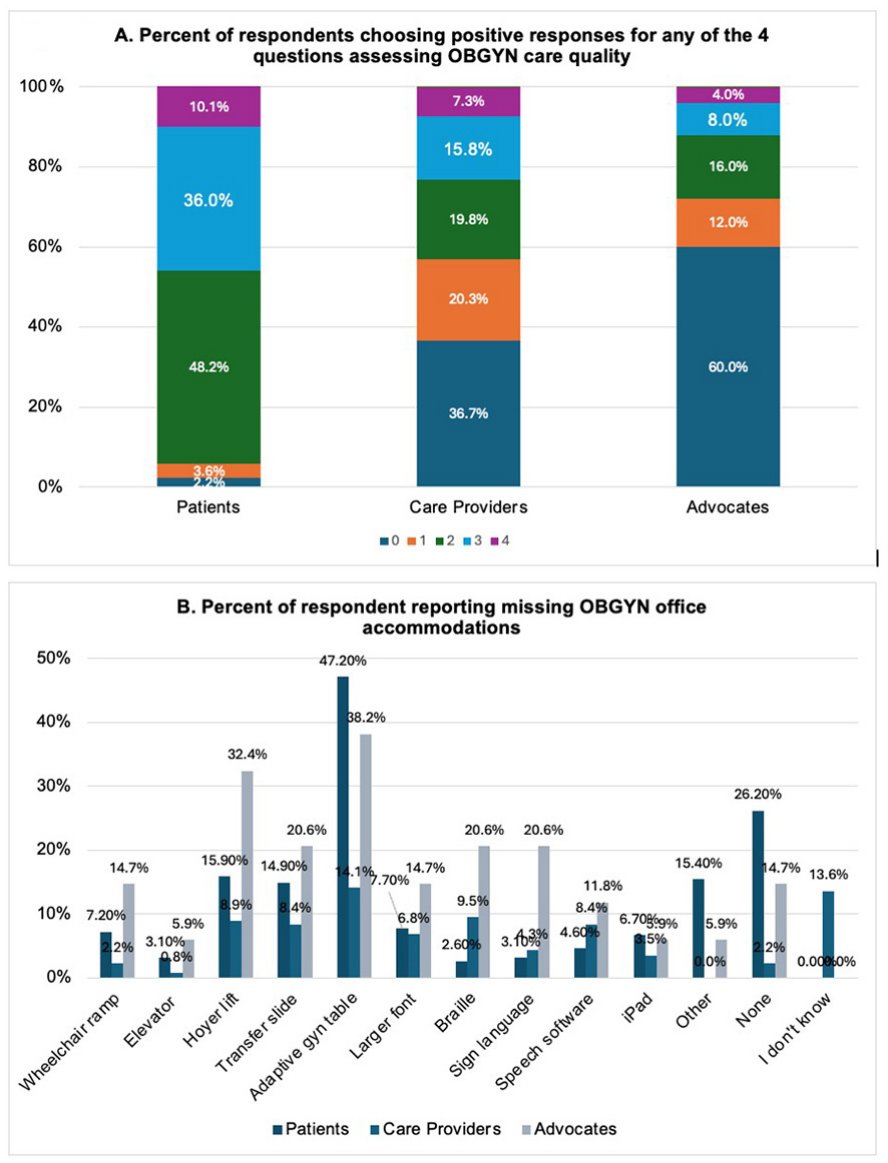
Perceptions of OB/GYN care quality and accommodation availability among patients, care providers, and advocates. **(A)** Proportion of participants responding positively to at least two of four care quality statements, stratified by stakeholder group. Significant differences were observed across groups (*p* < 0.001); 94.3% of providers, 42.9% of patients, and 28.0% of advocates responded positively. **(B)** Accommodations identified as missing from OB/GYN clinics by all participant groups. Height-adjustable examination tables and Hoyer lifts were most frequently cited as absent; over one-third of providers reported being unaware of what accommodations their offices needed.

Significant differences were also observed between patients, care providers and advocates in their subjective perceptions of OB/GYN care quality for patients with disabilities (Table 2). Overall, 94.3% (95% CI: 89.1–97.5%) of providers responded positively to at least two of the four care quality statements; only 42.9% (95% CI: 35.5–49.8%) of patients and 28.0% (95%CI: 12.9–44.4%) of advocates reached this threshold (*p* < 0.001) (Figure 3A). Less than half of patients agreed or strongly agreed that OB/GYN care teams make the effort to prioritize the comfort and safety of disabled patients (42.4%; 95% CI:35.5–49.8%) and to patients feeling heard and understood by their OB/GYN care teams regarding their disability-related needs (43.0%; 95% CI: 36.0–50.3%), while nearly all care providers (94.2%; 95% CI:89.1–97.5 and 95%; 95% CI: 90.0–98.0%, respectively) responded positively to these statements. Advocates provided fewer positive replies than both patients and providers (36%; 95% CI: 18.0–57.5 and 24%; 95% CI: 9.4–45.1%, respectively, *p* < 0.001 for both; Table 2). More than 75% of all participants agreed that OB/GYN care teams could be doing more for accessibility for patients, with no differences between the groups (*p* = 0.058; Table 2).

There were no differences between patients, providers and advocates on factors that positively and negatively impact care for patients with disabilities. Physical barriers – including inaccessible examination tables, absence of wheelchair scales and transfer equipment, inaccessible restrooms, inadequate parking, and narrow doorways – were cited as the primary factor negatively impacting OB/GYN care by approximately half (51.3%; 95% CI: 44.0–58.5%) of all participants. Over 30% of patients reported unpleasantness and social barriers as the most important factor negatively impacting OB/GYN care for disabled patients. Most participants (64.9%; 95% CI: 58.0–71.8% of all participants) believed that having disabled patients needs accommodated is what MOST positively impacts the quality of care for patients with disabilities. Providing a pleasant/friendly, warm environment was the second most common response (21.8%; 95% CI: 16.4–28.5% of participants).

Thematic analysis of open-ended responses from patients also reflected the importance of appropriate accommodations and relationships with care teams on perceptions of care quality for individuals with disabilities. Representative quotes included: “Every room should have a Hoyer lift if staff are not permitted to lift.”; “When you come into a welcoming environment, instead, this is an obstacle course.”; “Finally, I have a doctor who understands people's disabilities. Freedom: it means dignity and humanity.”; “The more comfortable you make the patient, the more they're actually able to collaborate and cooperate.” Additional themes included feeling rushed and the need for extended time for appointments to accommodate complex histories and physical limitations, the use of multiple communication modalities and sensory accommodations during examinations, procedures and informed consent, and care provider training. Safety concerns resulting from unskilled providers or unsatisfactory accommodations were also reported.

All participants felt that there were significant missing accommodations in OB/GYN clinics (Figure 3B). The most commonly missing accommodations identified were height-adjustable examination tables and Hoyer lifts (Figure 3B). Over one-third of providers were unaware of what accommodations their offices needed (Figure 3B). Fewer than 20% of providers felt that they were adequately trained in any of the accommodations. Few care providers (18.8%; 95% CI: 12.5–26.0%) reported receiving formal training in caring for patients with disabilities. Hoyer lift (21.1%) and height-adjustable examination table use (19.8%; 95% CI: 13.1–26.8%) were the most selected accessibility tools for which providers desired more training.

## 4 Discussion

This study represents one of the largest investigations of accessibility in OB/GYN care for patients with disabilities. Our novel triple-perspective methodology revealed critical gaps between patients, care providers and advocates in perceptions about the magnitude and impact of OB/GYN care disruptions for patients with disabilities. While less than half of all participants surveyed conveyed OB/GYN care disruptions due to unmet accommodation needs, more OB/GYN providers perceived gaps than patients and advocates. In contrast, OB/GYN providers' responses to the impact of care disruptions underestimated the duration of delays and unexecuted follow-up compared to patients and advocates. Care providers were more likely than patients and advocates to believe that OB/GYN care teams prioritize the comfort and safety of patients with disabilities, that patients feel heard and understood and that OB/GYN providers accommodate patients with disabilities well. Despite differences in perceptions about the impact of care disruptions and qualitative perceptions about patient experience, there was notable consensus that OB/GYN care teams could be doing more for accessibility for patients with disabilities.

While literature about typical wait times for rescheduling OB/GYN visits after canceled appointments is lacking, Kyllo et al. reported an average wait time of ~30 days for new patient appointments for general gynecology care (17). These findings suggest that provider reports of rescheduling patients in less that 2 weeks, may be underestimated. Finney et al. surveyed nearly 400 patients seen in two outpatient primary care clinics and gathered data demonstrating that patients with disabilities were less likely to be examined on an exam table and that patients who were not evaluated on a table had less favorable perceptions of their provider's bedside manner and were less likely to have positive perceptions of their provider's work compared to patients without disabilities. Devkota et al. (18) demonstrated that providers were motivated to provide quality care for women, while we showed that most providers believe that quality care is being delivered (19). Differing opinions about care quality and provider motivation between patients and providers in our study may result from the acknowledgment by providers in both studies that providers lack knowledge and skills about providing services (19).

Our study was not designed to acquire objective data on wait times, the performance of physical exams or provider experience treating patients with disabilities. However, our findings align with a smaller study by Sonalkar et al. (18) who interviewed 29 women with physical disabilities and 20 OB/GYN providers who had experience caring for patients with physical disabilities. They found that providers underestimated barriers and overestimated their ability to provide quality care (18, 20). Our study extends this work by quantifying gaps across a larger, self-selected, nationwide population with diverse disabilities and including advocates as a third perspective.

Our findings are particularly timely given the American College of Obstetricians and Gynecologists' recent Committee Statement No. 18 emphasizing the need for accessible obstetric and gynecologic care for patients with disabilities (4). Our study provides the evidence base to support implementation of professional recommendations and helps increase our understanding of where directed focus may be needed. Further studies integrating qualitative and measurable metrics are essential to enhancing gyn care for patients with disabilities (18, 20).

Inadequate provider skill and knowledge are indeed concerning. In our study, only 19% of care providers reported undergoing training in caring for disabled patients. This is consistent with another national survey demonstrating that approximately 17% of OB/GYNs had received any training in caring for patients with disabilities (21). Investing in provider competency has proven benefits on patient safety, patient and provider wellbeing, clinical efficiency and healthcare costs (21). Notably, patients, providers and advocates in our study agreed that appropriate accommodations, effective communication, and strong interpersonal relationships had the biggest impact on care for patients. Thus, while infrastructure improvements are necessary, addressing communication gaps is equally critical. Our findings align with existing literature demonstrating the universal importance of culturally responsive care, including respectful treatment, shared decision-making, and accessible health information (5, 6, 8, 10–16, 22–27).

The strengths of our study include a large, diverse, nationwide population encompassing multiple stakeholder perspectives using quantitative and qualitative metrics to assess opinions, behaviors, and trends. This evidence-based approach provided actionable guidance to improve accessibility and care quality while addressing the practical needs of both patients and providers. Our electronic format using QR codes and HTML links allowed for easy translation into adaptable formats to enhance accessibility and flexible time for survey completion. Our use of an on-line, open-access, opt-in, approach engaged participants across-disabilities, addressing a limitation in health disparities research that often segments disabilities into isolated categories and may overlook experiences of individuals with multiple disabilities.

Limitations include the cross-sectional design preventing causal inferences, and potential literacy and response bias from the web-based recruitment and reliance on self-reported data. This design also prevented an accurate assessment of the overall response rate. Our predominantly educated, English-speaking population suggests sampling bias, as national data indicate that less than 10% of patients with disabilities have a bachelor's degree or greater. Our convenience sampling approach may also introduce selection bias, as participants self-selected into the study and may not reflect the broader population of individuals with disabilities; findings should therefore be interpreted as indicative rather than fully representative of all patients, providers, and advocates. A critical limitation is the diversity of disability types represented: the majority of patients reported physical or cognitive disabilities, while individuals with sensory, intellectual, or complex communication needs were less represented. This limits the generalizability of findings across the full spectrum of disability experiences. While these limitations may alter the generalizability of our findings, the results provide valuable insight, highlighting key gaps in perceptions about accessibility and training that may exist more broadly.

## 5 Conclusions

Patients, providers and advocates acknowledge significant disruptions in OB/GYN care due to unmet accommodation needs, with critical gaps between patient, provider and advocate perceptions of the impact. All stakeholders agree that more needs to be done to improve accessibility for this vulnerable population. As healthcare systems focus on value-based care and health equity, addressing accessibility represents both a moral imperative and a practical necessity for improving health outcomes and reducing provider burden and global healthcare costs. This shared recognition that improvement opportunities exist provides a foundation for collaborative quality-enhancement initiatives across OB/GYN care systems.

## Data Availability

The original contributions presented in the study are included in the article/supplementary material, further inquiries can be directed to the corresponding author.
